# Sleep Apnoea Adverse Effects on Cancer: True, False, or Too Many Confounders?

**DOI:** 10.3390/ijms21228779

**Published:** 2020-11-20

**Authors:** David Gozal, Isaac Almendros, Amanda I. Phipps, Francisco Campos-Rodriguez, Miguel A. Martínez-García, Ramon Farré

**Affiliations:** 1 Department of Child Health, The University of Missouri School of Medicine, Columbia, MO 65201, USA; 2 Unitat de Biofísica i Bioenginyeria, Facultat de Medicina i Ciències de la Salut, Universitat de Barcelona, 08036 Barcelona, Spain; isaac.almendros@ub.edu; 3 CIBER de Enfermedades Respiratorias, 28029 Madrid, Spain; fracamrod@gmail.com; 4 Institut d’Investigacions Biomediques August Pi Sunyer, 08036 Barcelona, Spain; 5 Department of Epidemiology, University of Washington School of Public Health, Seattle, WA 98195, USA; aiphipps@uw.edu; 6 Epidemiology Program, Fred Hutchinson Research Cancer Research Center, Seattle, WA 98109, USA; 7 Respiratory Department, Hospital Valme (Seville, Spain), Institute of Biomedicine of Seville (IBiS), 41014 Seville, Spain; 8 Pneumology Department, Sleep-Disordered Breathing and Research Unit, Polytechnic and University La Fe Hospital, 46026 Valencia, Spain; mianmartinezgarcia@gmail.com

**Keywords:** sleep breathing disorders, malignancies, intermittent hypoxia, sleep fragmentation

## Abstract

Obstructive sleep apnoea (OSA) is a prevalent disorder associated with increased cardiovascular, metabolic and neurocognitive morbidity. Recently, an increasing number of basic, clinical and epidemiological reports have suggested that OSA may also increase the risk of cancer, and adversely impact cancer progression and outcomes. This hypothesis is convincingly supported by biological evidence linking certain solid tumours and hypoxia, as well as by experimental studies involving cell and animal models testing the effects of intermittent hypoxia and sleep fragmentation that characterize OSA. However, the clinical and epidemiological studies do not conclusively confirm that OSA adversely affects cancer, even if they hold true for specific cancers such as melanoma. It is likely that the inconclusive studies reflect that they were not specifically designed to test the hypothesis or because of the heterogeneity of the relationship of OSA with different cancer types or even sub-types. This review critically focusses on the extant basic, clinical, and epidemiological evidence while formulating proposed directions on how the field may move forward.

## 1. Introduction

In recent years, a theoretical assumption has been formulated and posited that obstructive sleep apnoea (OSA) may adversely affect the incidence and outcomes of cancer [1,2,3]. The biological framework for such putative associations is rather attractive, particularly in light of the extensive previous fundamental work linking hypoxia and cancer [4,5]. However, findings from epidemiological studies have been somewhat limited by the fact that most, if not virtually all, of the published studies to date were not specifically designed to address this relevant question. Nevertheless, the body of evidence that has accumulated to date has begun to unravel an understanding of several important pathophysiological mechanisms linking OSA and solid tumour biology. Furthermore, some of the epidemiological data have also enabled the realization that specific attributes of the tumour type as well as additional patient-related factors are critical operators, and ultimately determine whether an association between OSA and cancer incidence or outcomes will be either present or undetectable. Here, we will critically review the basic, clinical, and epidemiological studies on this topic, and formulate some proposals on how the field could move forward.

## 2. Biological Plausibility—Cellular and Animal Models

**Figure 1:**
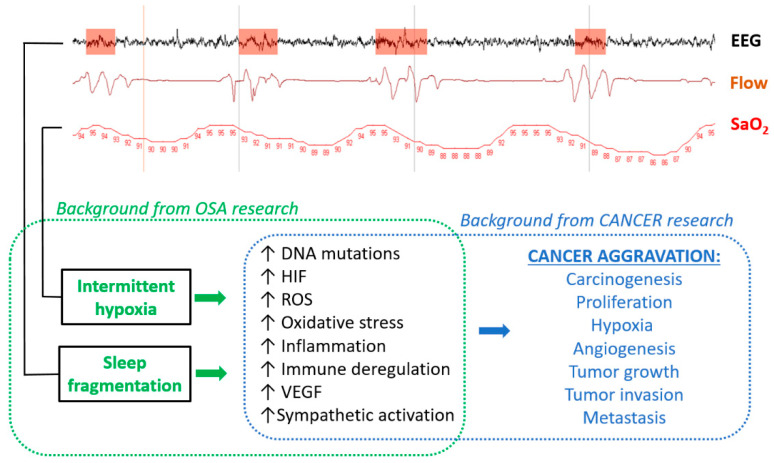
Diagram illustrating the biological plausibility of cancer aggravation by obstructive sleep apnoea (OSA). (**Top**) Example of the signals extracted from a routine polysomnographic study in a patient with OSA: an electroencephalography channel (EEG) showing arousals (orange); breathing flow assessed by nasal prongs (Flow), showing cyclic apnoeas and ventilation; and arterial oxygen saturation (SaO_2_), indicating intermittent hypoxemia (figures are SaO_2_ in %). A background of OSA research indicates that intermittent hypoxia and sleep fragmentation induce biological alterations, which, according to the background of cancer research, are modulators of cancer aggravation.

Proving that there is a cause–effect relationship between OSA and cancer is difficult since a considerable number of confounding variables are commonly present in patients suffering from both diseases [6]. However, analysis of biological plausibility using cell and animal models—a common approach in translational medicine—has so far provided some strong evidence of causality in the OSA–cancer relationship [7,8]. The experimental research has been focused on intermittent hypoxia (IH) and sleep fragmentation (SF) based on the fact that these two perturbations constitute the most salient hallmarks of OSA, and have been implicated in the other end-organ morbidities of the disease, while also being assumed to more directly impact on cancer progression (Figure 1) [3]. IH is elicited by the recurrent apnoeas/hypopnoeas originated by upper airway collapse during sleep. The ensuing arterial oxygen desaturation events are translated to all the patient’s tissues, triggering a wide-range cascade of cellular responses, including upregulation of hypoxia-inducible factors (HIF); oxidative stress; transcriptional factors underlying inflammation, such as nuclear factor kappa B (NFκB) and its downstream cascades; augmentation of reactive oxygen species (ROS); vascular endothelial growth factor (VEGF) expression; increased DNA mutations; and immune deregulation (Figure 1) [1,3,9,10,11]. SF in OSA is caused by the recurrent micro-arousals, usually terminating the apnoeic/hypopnoeic events. This OSA-specific type of sleep architecture disruption also triggers relevant biological responses, such as increases in sympathetic nervous system tone and reactivity, augmentation of ROS, inflammation, and immune deregulation [1,12,13]. Remarkably, these biological end-products of both IH and SF are also the main operators in malignancies, particularly carcinogenesis, cancer cell proliferation, and angiogenesis, resulting in an increased tumour growth rate, and cancer cell migration, leading to tumour invasion, extravasation, and metastasis (Figure 1). Therefore, our current knowledge of the pathophysiology of cancer and OSA provides biological plausibility to the hypothesis that this sleep breathing disorder can initiate and/or aggravate malignancies.

**Figure 2:**
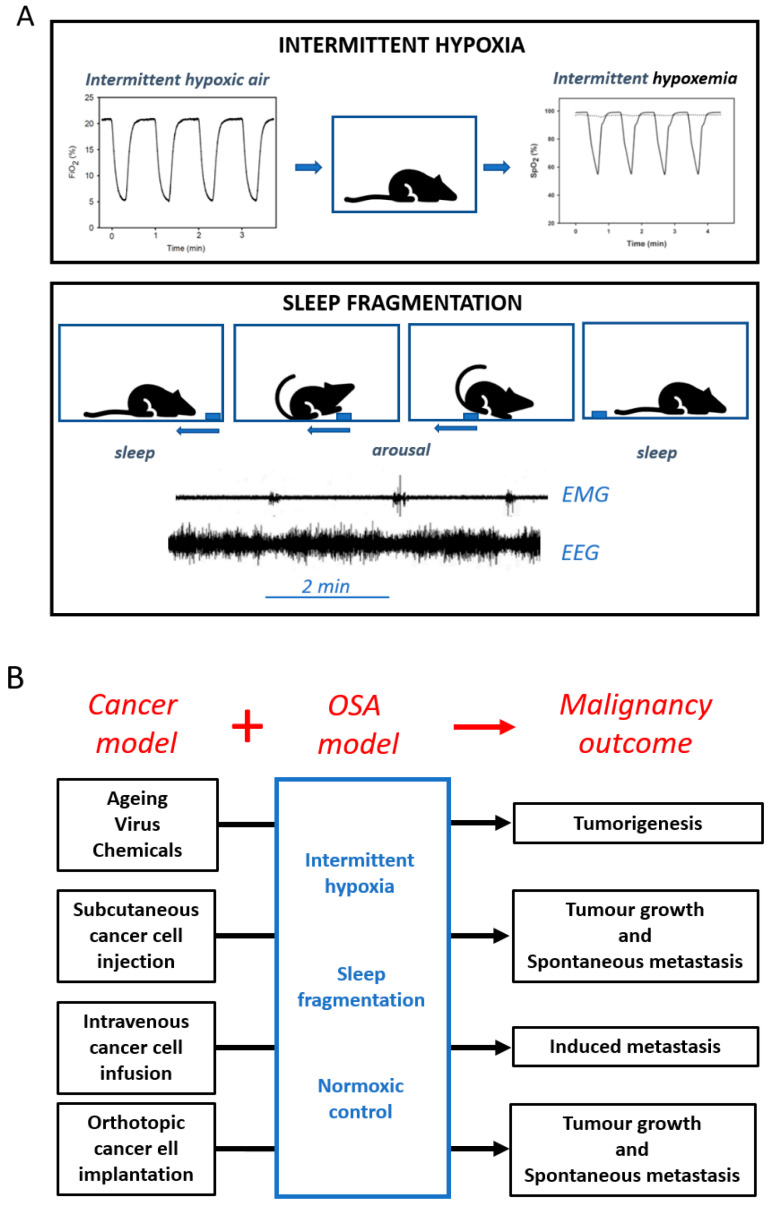
(**A**) Diagrams of the experimental settings to apply intermittent hypoxia (**top**) and sleep fragmentation (**bottom**) to rodents. Air with cyclic O_2_ fraction (FiO_2_) in the animal cage induces recurrent hypoxemia (SaO_2_). Smooth cyclic movement of a bar (blue) in the cage ground induces cyclic arousal, as reflected by electromyography (EMG) and electroencephalography (EEG) signals in the mice. (**B**) Experimental application of the obstructive sleep apnoea (OSA) challenges (IH: intermittent hypoxia, SF: sleep fragmentation) and control normoxia to cancer models of tumorigenesis (spontaneous by ageing or induced by virus/chemicals) and tumours induced by subcutaneous, intravenous, or orthotopic cancer cell application.

Studies using cell culture models have supported some of the basic mechanisms involved in the tumour-enhancing effect of IH—the only OSA challenge directly applicable to cells—on a variety of cancer cell types originating from different organs (e.g., breast, cervical, gastric, melanoma, pancreas, lung, and colorectal), mainly showing increased tumour cell proliferation and up-regulation of HIF and NFκB activity [14,15]. Moreover, co-cultures of lung or melanoma cancer cells with preconditioned tumour-associated macrophages from animals subjected to IH or SF reveal deregulation of the immune system, leading to enhanced cancer progression [1]. Moreover, research in animal models―which integrate physiological responses at the multi-organ level—has provided evidence on how IH and SF may enhance malignancy progression [16,17]. IH exposures have been implemented by cyclically changing the oxygen composition of the air breathed by rodents, thereby inducing recurrent hypoxemia (Figure 2) with the amplitude, duration, and frequency mimicking those experienced by patients with different OSA severity [18]. SF has been induced in rodents by mechanical approaches that very briefly and without stress arouse the animals at frequencies similar to those in OSA patients (Figure 2) [19]. Interestingly, both IH and SF have been applied for extended periods of time spanning weeks to months, usually consisting of several hours daily during the usual sleep cycle, and with the animals placed in a cage allowing them to freely move, access food and water ad lib, and socially interact. These realistic OSA mimicking paradigms have been implemented in conventional rodent cancer models to investigate potential increases in tumorigenesis in intact animals [20] or explore alterations in tumour properties such as growth, local invasion or metastasis in animals subjected to an exogenous tumorigenic challenge (Figure 2). The interest in combining OSA and cancer models resides in the potential direct observation of the effect of IH/SF on cancer biology in otherwise healthy animals, and thus avoid the many confounding factors that are usually present in OSA patients [6]. Alternatively, some such confounding factors may be selectively introduced, to allow for studies of the interactions between, for example, IH and obesity [21], IH and ageing [22], and IH and menopause [23]. Table 1 presents a summary of the findings reported by 30 studies in animal models that investigated whether OSA boosts cancer malignant properties [13,16,17,21,22,23,24,25,26,27,28,29,30,31,32,33,34,35,36,37,38,39,40,41,42]. The overall summary is reflected by the conclusion that both IH and SF increase tumorigenesis, tumour growth, invasion/metastasis, and angiogenesis.

Despite the rather compelling findings summarized in Table 1, some important issues must be pointed out. First, most data from animal models came from experiments carried out in two types of murine cancer (lung and melanoma) that only included a very restricted number of cancer cell types, such that the transferability of these findings to human cancers remains uncertain. Second, most studies were focused only on tumour growth, while invasion and metastasis were not as frequently included as outcome variables. Third, most reported data originate from IH exposures, with the effects of SF being much less frequently investigated (3 out of 30 studies). Remarkably, there are no data on the effects of concurrent application of IH and SF to better mimic the clinical phenotype of OSA patients during sleep. Fourth, in most cases the animals were subjected to IH/SF challenges that mimicked rather severe OSA, which is reasonable to highlight a phenomenon, but may not represent the much greater spectrum of OSA severity and patient phenotype heterogeneity. Fifth, the virtually absent reports indicating that cancer aggravation fails to occur following IH/SF may reflect publication bias affecting negative results. Notwithstanding the limitations inherent to experimental models and the precautions required in translation of results from animal models to patients, the currently available data from experimental research provide solid biologically based evidence to support the hypothesis that OSA contributes to the initiation and/or exacerbation of some types of cancer.

## 3. Not All Tumours are Born Equal—Differential Susceptibility to Intermittent Hypoxia

As will become apparent in the subsequent paragraphs, most epidemiological studies have explored “cancer” as the single outcome variable, rather than focus on individual tumour types, and use very specific inclusion criteria. Such an approach and the consequent disparate findings from such studies strongly indicate that a substantial degree of heterogeneity in the response to either IH or SF is present among tumour cells. To address this issue, Marhuenda and colleagues have recently reported on the vastly divergent responses of human histological subtypes of non-small cell lung cancer, namely, specific cells lines of adenocarcinoma and squamous cell carcinoma [45]. Furthermore, the characteristics of the hypoxic stimulus and its interactions with the cancer cell of interest may impose fundamentally different responses that may or may not lead to alterations in tumour cell phenotypic properties [5]. Considering the extraordinary complexity of the interactions between established and predictable hypoxic profiles and tumour cell biological pathways, it should not be surprising that the individual signature of the hypoxic and sleep profiles in each patient with OSA will elicit markedly divergent responses, and that the complexity of such responses will be further compounded by the intrinsic properties of the cancer cell of interest and its evolutionary genetic, epigenetic, and phenotypic variations over time [46].

## 4. Potential Mechanisms and Immunological Considerations—Effects of Obesity and Aging

**Figure 3:**
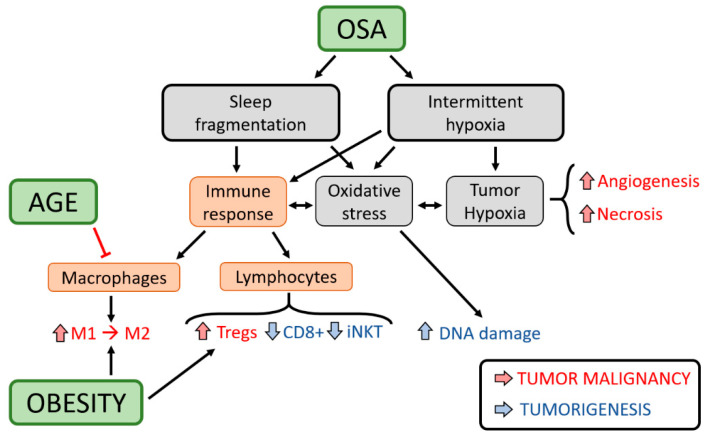
Potential mechanisms and immunological alterations in the interaction between OSA and cancer (see the text for an explanation).

Since the first studies linking OSA and cancer were published in 2012 [33,47,48], the number of translational and clinical studies aiming to explore the veracity of this association and the potential mechanisms underlying has steadily increased. Considering that the presence of a hypoxic environment has been widely associated with increased tumour malignancy and poor prognosis [49], several initial hypotheses tested included (i) assessing whether the oxygen desaturation characteristics of OSA, induced by exposing mice to IH, are transmitted to the tumoral tissue; and (ii) to elucidate how tumour growth is affected by this stimulus. As expected, oxygen partial pressure (PO_2_) measurements carried out in a mouse model showed that exposures to IH induced recurrent severe reductions in tissue PO_2_ in well-irrigated areas of primary melanoma tumours [33] (Figure 3). This reduction in tissue PO_2_ promoted increased VEGF expression, tumour growth, and larger necrotic areas. However, additional studies suggest that the effects of IH at the cellular level can vary among different cancer types, even between different histological types of lung cancer [45]. In particular, data from an animal model of multiple myeloma [42] suggests that the response mechanisms to intermittent hypoxia can differ between non-solid and solid tumours. Thus, differential responses of cancer cells to hypoxia may be consequent to existing or emerging specific mutations or genetic signatures, and these issues still need to be thoroughly investigated.

In addition to the direct effects of hypoxia on cancer cells, a higher cancer incidence in OSA could be mediated by alterations in the immune system (Figure 3). Immune cells play a major role in the surveillance against cancer, but these functions can be altered under a variety of circumstances and facilitate tumour escape. Experimental data have shown that both IH and SF mimicking OSA promote changes in the immune system, leading to systemic and local inflammation and oxidative stress. These alterations may compromise immune surveillance, and account for the increased tumorigenesis observed in mice subjected to IH, and also the increased cancer incidence reported in some human cohorts [2]. Among these alterations, some include (i) IH-induced HIF activation can up-regulate the co-inhibitory axis of the programmed cell death receptor 1 and its ligand (PD-1/PD-L1), thereby conferring a lower proliferative and cytotoxicity capacity to T cells [32,50]; (ii) IH and SF chronic exposure reduce tumour CD8+ T cytotoxic function [13]; and (iii) OSA has been associated with impairments in invariant natural killer (iNKT) cell maturation and cytotoxicity, as well as iNKT apoptosis [51] (Figure 3). Interestingly, these alterations in iNKT cells can be partially reverted by CPAP treatment [51]. Moreover, CPAP treatment was able to reduce the expression of enriched gene sets in peripheral blood leukocytes involved in tumorigenesis [52]. Once the tumour is established, cancer cells can disable and re-educate some immune components to facilitate tumour progression. At this stage, tumour-associated macrophages (TAMs) are considered one of the major players in this transition, changing their function from an anti-tumoral (M1) to a pro-tumoral (M2) phenotype. In animals exposed to IH and SF, a higher infiltration of M2 macrophages and regulatory T lymphocytes (Tregs), potent immune suppressor cells, were found in the tumour stroma [16,17] (Figure 3). Interestingly, isolated TAMs from these tumours fostered enhancements in lung cancer malignancy in a co-culture system in the absence of any other stimuli, thereby attesting to the potential involvement of IH/SF-induced modifications in the immune system in contributing to accelerated cancer progression. To date, the adverse cancer outcomes observed under IH/SF seem to be mediated, at least in part, by changes in the Toll-like receptor (TLR)-4 [16], cyclooxygenase (COX)-2 [29], VEGF [43], metastasis-related matrix metalloproteinases (MMPs) [43], nicotinamide adenine dinucleotide phosphate (NADP)H oxidase type 2 [22], and m6A demethylase ALKBH5 pathways [44].

Taking into account that OSA prevalence increases with obesity and with ageing, and since both obesity and advanced chronological age are known to modulate cancer behaviour, they are currently viewed as two of the main confounding factors in the putative relationship between OSA and cancer [2]. In this context, there are epidemiological and clinical data revealing that the strongest association occurs among young and non-obese patients, and that such associations weaken with the presence of obesity and older age [2]. Obesity not only represents a well-described risk factor of OSA, but also the concurrent presence of OSA in obese patients can foster the risk for development of metabolic syndrome [53]. In parallel with the increased risk of tumorigenesis due to the chronic inflammation induced by OSA in adipose tissues, the recruitment/proliferation of inflammatory macrophages within these tissues can also serve as a depot of immune cells facilitating tumour progression. Indeed, macrophages in the adipose tissues of mice exposed to IH increase their migration and infiltration toward the tumour, and ultimately increase cancer cell malignant properties [25] (Figure 3). However, how high fat diet in OSA, IH/SF-induced metabolic components, or IH/SF-induced microbiome alterations can modulate tumour progression is still unknown. At the cellular level, IH increases MCF7 breast cancer survival and induces the Warburg effect through upregulation of the expression of genes involved in the glycolysis [54]. Gut microbiota from OSA patients can also activate the HIF-1α expression and STAT3 pathway in Immorto-Min colonic epithelial cells [55].

In the context of ageing, the associations between OSA with some cancer types showed differences as a function of age [2]. Considering that ageing may downregulate the immune responses to injurious challenges, it is plausible that the age differences in the OSA-cancer associations could be mediated by a reduced inflammatory response in aged patients with OSA. Recent works are supportive of this notion. First, a recent study revealed that the increased recruitment and polarization of TAMs toward a pro-tumoral phenotype induced by IH in young mice does not seem to occur in older animals, resulting in the absence of detectable changes in tumour growth [22] (Figure 3). Second, the PD-L1 upregulation reported in OSA patients and mice subjected to IH occurs mainly in young individuals possibly due to impaired oxygen sensitivity in older ones [56]. Third, it has been shown that hormone alterations induced by ovariectomy promote an accelerated lung cancer progression in a mouse model of menopause [23]. Although clearly more studies are needed to better understand the role of the immune system and the interactions with obesity and age in the relationship between OSA and cancer, currently available experimental evidence is essential for the design and development of future clinical/epidemiological studies.

## 5. Melanoma as the Prototype

Although it accounts for only 5% of all cutaneous malignancies, melanoma is one of the most aggressive forms of cancers with a worldwide incidence of 3/100,000 inhabitants. Among the risk factors of melanoma, UV exposure, tanning-bed use, fair skin, hair colour, and family and personal history are well recognized. However, very little is known about the factors associated with a higher melanoma aggressiveness, which is crucial to develop new treatments options [57].

As mentioned in previous sections of this review, there is biological plausibility to link OSA and its main components, i.e., IH and SF, with increased prevalence, incidence, and aggressiveness of several types of cancers. However, this association seems to be modulated by the location and histological type of the tumours [1,2,3]. As discussed above, we should therefore assume that not all cancerous cells will mount similar responses (and hence share similar pathophysiological pathways) to the ongoing changes in oxygenation, immune deregulation and alterations in sleep structure that characterize OSA [8]. In spite of the limited literature about the relationship between OSA and different types of cancer, cutaneous melanoma has probably received the greatest level of interest, as illustrated not only by the large number of studies performed in animal models, but also clinical and epidemiological studies in humans [58]. Moreover, notwithstanding inherent limitations, the vast majority of these studies concluded that a significant relationship was present between the prevalence, incidence, or aggressiveness of melanoma and different measures of OSA severity [58]. Table 2 shows the baseline characteristics and findings of the main clinical and epidemiological studies published to date.

The largest study that analysed the relationship between OSA and some types of cancer, including melanoma, was published by Gozal et al. The authors evaluated a cohort of 5.6 million individuals with OSA with a follow up of 4 years, and compared them with matched control groups. Patients with a diagnosis of OSA had 1.14 (95%CI 1.10–1.18; *p* < 0·0001) increased probability to be subsequently diagnosed with melanoma compared to the non-OSA group at the end of the study. Moreover, the OSA group showed a lower risk of metastatic spread, although the risk of death caused by melanoma was not increased [60]. Similarly, Sillah et al. recently analysed a cohort of 34,402 OSA patients with a follow-up of 5.3 ± 2.0 years linked to a population-based cancer registry. At the end of the study, the adjusted incidence of melanoma was higher in the OSA group (incidence ratio 1.71, 95%CI 1.42–2.03), with similar risk elevations in men and women [61]. However, not all clinical studies found an association between OSA and a higher incidence of melanoma. For example, Cohen et al. evaluated three prospective US cohorts accounting for 2,301,445 person-years of follow-up, and did not find any relationship between snoring, clinical risk of sleep apnoea, or sleep duration and the risk of melanoma. However, this study presented important limitations since it was not based on sleep study diagnosis, but rather relied on self-reported information and retrospective recall of OSA diagnosis by physicians [59].

As commented, although a significant increase in incidence (but not aggressiveness) of melanoma emerged in OSA patients, the studies suffer from several important limitations—namely, their retrospective nature and the use of health administrative databases with a lack of analysis of important confounders (especially the presence of obesity)—thus precluding robust conclusions. In order to avoid these limitations, a pilot study in 56 patients [62] and, subsequently, a major prospective multicentre study that included 443 newly-diagnosed melanoma patients, were performed in Spain, and aimed to analyse the relationship between the presence and severity of OSA and aggressiveness markers of melanoma [63]. The results of these two prospective studies not only confirmed the previous assumptions but further showed that, compared to patients in the lower tertiles of OSA severity, those in the upper tertiles were 1.94 (95%CI, 1.14–3.32) and 1.93 (95%CI, 1.14–3.26) times more likely, respectively, to present with aggressive melanoma as defined by a Breslow index >1 mm, after multiple adjustments for confounders. Interestingly, the relationship between OSA and melanoma aggressiveness was stronger in patients <56 years, in parallel with similar patterns in studies on melanoma incidence [61], and as has previously been reported in other clinical [47,64], animal models [22,56], and biomarker [56,65] studies. There are still more questions than answers on the relationship between OSA and cutaneous melanoma, particularly the necessity to find accurate biomarkers, such as circulating microRNAs, to confirm a causal relationship between these two disorders, or determine the role of OSA and CPAP treatment in the long-term outcomes of melanoma and response to current treatments [58].

## 6. What about Other Specific Cancers?

Considering that the effect of IH and SF may differ depending on the susceptibility and adaptive mechanisms of each specific organ and type of cancer cell, it can be hypothesized that the deleterious effect of OSA on both the incidence and prognosis of cancer will vary depending on the specific cancer site and the histological type of tumour. Thus, the evidence observed for one specific cancer cannot be extrapolated to others. Unfortunately, except for cutaneous melanoma, the evidence about this relationship in other tumours is scarce and biased by substantial methodological pitfalls. Although some epidemiological studies have tried to address the association between OSA and specific cancer sites, the information obtained from these cohorts is incomplete and affected by different kinds of limitations, mainly the source of information and that they were not designed to address this association. For example, the information retrieved from a nationwide health insurance database showed that patients with OSA had an increased risk of incident pancreatic, kidney, and melanoma cancers, but a lower risk of colorectal, breast, and prostate cancers [60]. The presence of OSA, however, was not associated with poorer cancer outcomes. In another retrospective case-cohort study, the proportion of patients with severe OSA was higher among individuals with cancers of the prostate, lung, and kidney, as well as melanoma, than among those in a randomly sampled sub-cohort of OSA patients, suggesting that more severe OSA would increase the risk of developing certain types of cancers [61]. However, very few studies have been devoted to investigating the association between SDB and specific cancers [66,67,68,69,70,71,72,73,74,75,76,77,78,79,80].

Two of the more common cancers, lung and breast, have also attracted interest. The only study designed to investigate the association between OSA and breast cancer reported an OSA prevalence of 51.8% in 83 consecutive middle-aged women diagnosed with breast cancer [68]. No associations were found, however, between the OSA severity measures such as the apnoea–hypopnoea index (AHI), the time spent with oxygen saturation <90% or the oxygen desaturation index, and different markers of severity of breast cancer or any molecular subtype of breast cancer. Two retrospective studies that have used data from national health insurance databases have observed an association between OSA and incident breast cancer [66,69]. Studies on lung cancer have also reported prevalence rates of OSA between 42 and 49% [70,71]. A study with 302 subjects that combined two cohorts, one that investigated OSA prevalence in lung cancer patients and another that assessed OSA in subjects who participated in a lung cancer screening program, showed that the AHI and oximetric parameters were independently associated with the presence of lung cancer [70]. Two other studies reported controversial results about the role of OSA on cancer progression [72,73]. Reports on other cancer locations, such as colorectal, prostate, kidney, pancreas, lymphoma, and primary central nervous system cancers are anecdotal and, as previously stated, limited by methodological issues (Table 3) [74,75,76,77,78,79,80].

The shortage of studies focused on specific cancer sites, the small sample sizes, and the limitations associated with methodological issues have biased their potential findings and preclude drawing any strong conclusions on the potential associations between OSA and the incidence or prognosis on any organ-specific cancer, or histological cancer type. Well-designed studies are needed to clarify whether OSA impacts on specific cancers or tumour cells in addition to cutaneous melanoma.

## 7. Summary of Epidemiological Evidence—An Ocean of Confusion

As described in previous sections, multiple epidemiologic studies have previously attempted to characterize the relationship between OSA and cancer, often with conflicting results. Initial community-based longitudinal studies on this topic focused on cancer overall, noting a 3.4 to 4.8-fold elevation in cancer mortality rates among those with OSA [48,81]. Several subsequent studies similarly reported elevated cancer incidence overall among those with vs. without OSA [47,61,82,83,84].

**Figure 4:**
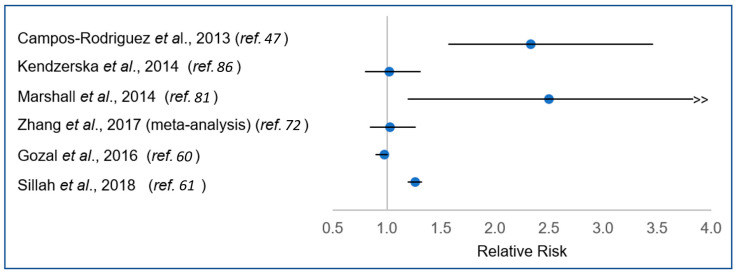
Relative risk for association between obstructive apnoea (OSA) and cancer incidence in previous epidemiologic studies.

However, the largest studies to date, including a meta-analysis, have found no significant association between OSA and cancer overall (Figure 4) [60,85,86]. With the exception of studies focused on cutaneous melanoma, which have fairly consistently indicated an increased risk or more advanced pathology associated with OSA [60,61,62,63], studies examining specific cancer sites have been limited and inconclusive [60,61,66,69,70,71,72,73,74,75,76,77,78,79,80,83] (Table 2 and Table 3). Thus, despite strong biological plausibility for an association of OSA with cancer aetiology and progression, and sound supporting laboratory evidence, epidemiologic studies remain inconclusive.

Several methodologic considerations and limitations likely underlie the observed differences across existing epidemiologic studies, highlighting the complexity of the OSA–cancer relationship. Key among these considerations is the fact that cancer is a heterogeneous disease. As such, cancer at different anatomic sites and/or with different pathologies may plausibly be differentially related to OSA. Thus, any true cancer’s site-specific effects are likely to be obscured in studies examining associations of OSA with overall cancer incidence or mortality, by virtue of combining those associated cancers with cancers from other sites not associated with OSA. The resulting impact of this combinations of effects may plausibly vary across study settings based on the distribution of the site-specific cancers.

In order to maximize sample size and facilitate more sensitive cancer site-specific analyses, a number of epidemiologic studies have leveraged large clinical or administrative databases, identifying individuals with OSA via recorded diagnostic codes [60,61,66,69,72,76,78,79,80,82,83,86]. While highly valuable, the use of such resources presents further potential for bias. In particular, administrative and medical records contain only limited information on a subset of relevant confounders, with information on obesity and smoking status (i.e., two especially important confounders frequently unavailable). Failure to adequately control for these confounders adversely affects the interpretation of the observed results, and may have different effects across studies depending on the underlying distribution of these risk factors in diverse study settings.

Reliance on administrative databases for identifying individuals with OSA also introduces an opportunity for selection bias and misclassification of exposure status. Specifically, given that 80–90% of adults with OSA are likely undiagnosed [87], any “unexposed” comparison group defined based on the absence of an OSA diagnosis is likely to include a non-negligible number of individuals who truly have existing, undiagnosed OSA. This problem may be particularly pronounced in clinical settings, in which the patient population is more likely to have risk factors predisposing to OSA (e.g., obesity). Conversely, the subset of individuals with OSA who do receive a clinical diagnosis is likely not a representative sample of the overall OSA-affected population. Studies using objective measures to ascertain OSA status in a community setting [48,81] would avoid these limitations; however, such studies are resource-intensive to conduct and, thus, limited in sample size.

Regardless of the study design or setting, all epidemiologic studies of OSA and cancer face the common challenge of establishing temporality. OSA and cancer are both chronic diseases. For both conditions, the time from disease initiation to diagnosis may span several years and, in the case of cancer, even decades. In order for the physiologic effects of OSA to impact upon the aetiology of cancer, OSA must be present (if not diagnosed) several years prior to the cancer diagnosis. Even if the role of OSA is restricted to cancer promotion and progression, it must be present at a time during the natural history of cancer at which it could plausibly have such effects. This temporal sequence is challenging, if not impossible, to determine. Given the variability across epidemiologic studies with respect to study design (i.e., retrospective vs. prospective, inclusion of different cancer sites) and the duration of the follow-up, it is probable that at least some of the observed variability between studies stems from differences in the relative timing of exposure and outcome. Despite these methodological challenges, and the inconsistency of the studies to date, there remains a critical need for high-quality epidemiologic studies to clarify the relationship of OSA with specific forms of cancer.

## 8. Where do We Go from Here?

The current evidence presented in this review places the highly compelling findings in biological studies as drivers of the push and need for sound and well-designed clinical and epidemiological studies (Table 4). Prospective multicentre clinical studies will be required to conduct careful phenotyping of sleep along with state-of-the-art characterization of the specific malignancy, and further track multidimensional and multi-omics parameters of cancer activity and aggressiveness. Parallel mechanistic studies in cell and animal models will need to carefully demarcate the unique experimental exposure profiles to IH or SF and expand the repertoire of the cancer lines for any specific type of cancer, while carefully addressing the fundamental questions as to the role of OSA in tumorigenesis, tumour properties and host responses, and their response to therapy. In other words, the journey is long, and we are just at the beginning.

## Figures and Tables

**Table 1:** Summary of animal model research focused on the effects of OSA on cancer aggravation.

Cancer Type	IH N Pub (References)	SF N Pub (References)	Tumour Growth	Invasion/Metastasis	Angiogenesis	Carcinogenesis
Melanoma	8 [17,21,23,33,34,35,36,37]	0	↑ 8/8	↑ 3/3	↑ 2/2	―
Lung	13 [13,17,20,22,25,26,27,29,30,31,32,43,44]	3 [16,24,28]	↑ 13/13	↑ 13/13	↑ 1/1	↑ 2/2
Kidney	1 [38]	0	↑ 0/1	―	↑ 1/1	―
Breast	2 [39,40]	0	↑ 2/2	↑ 2/2	―	―
Colon	2 [36,41]	0	―	―	―	↑ 2/2
Myeloma	1 [42]	0	↑ 1/1	―	―	―

Intermittent hypoxia (IH) and sleep fragmentation (SF): OSA challenge applied to the animals. N Pub: number of published papers; ↑ *x/y*: outcome increase observed in *x* studies out of the *y* in which it was assessed; ―: outcome not reported. Green/red indicate that the results did/not confirm the hypothesis that the OSA challenge aggravates cancer.

**Table 2:** Melanoma: most relevant clinical and epidemiological studies on its association with sleep-disordered breathing.

Author (Year) (Reference)	Design	Patients	Cancer Outcome	Main Results
**Epidemiological-based studies**				
Cohen (2015) [59]	Epidemiological study	Three prospective cohorts (178,633 subjects with 880 patients with melanoma)	Incidence	**Incidence**: No association between snoring, sleep duration or OSA and risk of incident melanoma
Gozal (2016) [60]	Epidemiological Case-control study	5.6 million subjects (with 1.7 million of OSA patients and 19,927 of melanoma patients) Health insurance database	Incidence Aggressiveness	**Incidence**: Higher in OSA patients **Aggressiveness**. No relationship between OSA and risk of metastasis
Sillah (2018) [61]	Epidemiological Retrospective study	34,402 patients with sleep apnoea (129 patients with melanoma)	Incidence	**Incidence**: Higher in OSA patients Association more prominent in patients <60 years and men
**Clinical-based studies**				
Martinez-Garcia (2014) [62]	Multicentric, prospective	56 patients with melanoma	Prevalence Aggressiveness	**Prevalence**: AHI ≥ 15: 30.3%. **Aggressiveness**. AHI and Tsat90% were predictors of fast-growing melanoma
Martinez-Garcia (2018) [63]	Multicentric, Prospective	443 patients with melanoma	Aggressiveness	**Aggressiveness**: AHI or DI4% values were associated with some markers of melanoma aggressiveness. Association more prominent in patients < 56 years and Breslow index > 2 mm

OSA: Obstructive sleep apnoea; Tsat90%: Night-time spent with an oxygen saturation below 90%; DI4%: Desaturation index at 4%; AHI: Apnoea-hypopnoea index; SDB: Sleep-disordered breathing.

**Table 3:** Non-melanoma malignancies: most relevant clinical and epidemiological studies on their association with sleep-disordered breathing (SDB).

Author (Year) (Reference)	Design and SDB Assessment	Patients	Main Outcomes
**Breast cancer**			
Chang (2014) [66]	Retrospective study. Data were retrieved from a national health insurance database. SDB was diagnosed based on ICD-9 codes Subjects were followed for 5 years.	846 women with SDB and 4230 age-matched control women without ICD-9 codes corresponding to SDB	-A diagnosis of SDB was associated with increased risk of having a diagnosis of breast cancer, after a 5 years follow-up, compared to the control group (adjusted HR 2.09; 95% CI 1.06–4.12).
Phipps (2016) [67]	Longitudinal study. SDB was not assessed by objective methods. Sleep characteristics were recorded at the time of enrolment.	6825 postmenopausal women with diagnosis of primary breast cancer during follow-up from the Women’s Health Initiative cohort.	-Women who reported at enrolment a sleep duration ≤6 h/night combined with frequent snoring (≥5 nights/week) had substantially poorer breast cancer survival than those reporting neither (HR 2.14, 95% CI 1.47–3.13).
Campos-Rodriguez (2018) [68]	Cross-sectional study specifically designed to address the association between SDB and breast cancer. Women underwent a home respiratory polygraphy. SDB was defined as an AHI ≥ 5.	83 consecutive women <65 years with a diagnosis of primary breast cancer.	-The prevalence of SDB was 51·8%, with a median (IQR) AHI of 5.1 (2–9.4) and ODI4 of 1.5 (0.5–5.8). -No associations were found between AHI or oximetric parameters and different markers of severity of breast cancer, including Ki67, Nottingham histologic grade, tumour stage, absence of hormone receptors, and molecular subtypes of breast cancer.
Choi (2019) [69]	Retrospective study. Data were retrieved from a national health insurance database. SDB was diagnosed based on ICD-10 codes Subjects were followed for 3.7 ± 2.3 years.	45,699 women >20 years with SDB and 228,502 age-matched control women without ICD-10 codes corresponding to SDB	-The incidence of breast cancer among women with OSA was significantly higher than that among the controls (HR 1.20, 95%CI 1.04–1.39). -The incidence of breast cancer was higher among patients aged ≥65 years (HR 1.72; 95%CI 1.10–2.71).
**Lung cancer**			
Seijo (2019) [70]	Cross-sectional study. Participants underwent a home respiratory polygraphy. SDB was defined as an AHI ≥ 15.	302 individuals from 2 cohorts: SAIL cohort, which investigated SDB prevalence in lung cancer patients, and SAILS cohort, which assessed SDB in subjects participating in a lung cancer screening program	-The prevalence of SDB was 42%. -Lung cancer was 8% more prevalent in patients with an AHI ≥ 15, compared to those with an AHI < 15. -After adjustment for potential confounders, AHI, nocturnal hypoxemia, including time spent below 90% oxyhaemoglobin saturation, and 3% oxygen desaturation index were significantly associated with lung cancer.
Dreher (2018) [71]	Cross-sectional study. SDB was assessed by means of a type IV device (two-channel screening system). SDB was defined as an AHI ≥ 5.	100 patients with newly diagnosed lung cancer from 3 German centres.	-The prevalence of SDB was 49%, with 32% showing mild SDB (AHI5-14.9) and 17% showing moderate-to-severe SDB (AHI ≥ 15). -The median (IQR) AHI of 7.7/h (5.4–10.4) and oxygen desaturation index of 8.5 (4.2–13.4).
Li (2017) [72]	Retrospective study. The method to investigate SDB is not clarified. SDB was defined as an AHI ≥ 5.	43 consecutive patients >18 years with concurrent SDB and lung cancer. Patients were obtained from an electronic hospital database. Patients treated with CPAP were excluded.	-Patients with moderate or severe SDB (AHI 15–29.9 and ≥30, respectively) and lung cancer had lower survival than those with only mild SDB (AHI5–14.9).
Liu (2019) [73]	Case-control study. 1-year follow-up SDB was assessed by respiratory polygraphy, although the threshold to define SDB is not provided.	45 patients with primary lung cancer suitable for surgical resection and 45 age and sex-matched controls.	-Patients with lung cancer had statistically significant greater risk of SDB, as well as more hypoxemia, time spent with O2 saturation <90% and higher ODI compared to the matched control group. -The SDB and control groups had similar risk of recurrence, metastasis or mortality after a 1-year follow-up.
**Colorectal cancer**			
Zhang (2013) [74]	Prospective cohort study. SDB was not assessed by objective methods. Sleep duration and snoring was recorded at the time of enrolment. Cancer diagnosis were obtained from medical records.	30,121 men in the Health Professionals Follow-up Study and 76,368 women in the Nurses’ Health Study	-The presence of long sleep duration (≥9 h/day) associated with regular snoring (defined as snoring at least few days per week) was associated with incident colorectal tumours in both men and women, compared to individuals who slept an average of 7 h (men: HR 1.80, 95% CI 1.14–2.84; women HR 2.32, 95% CI 1.24–4.36). -Short sleep duration (<5 h/day) was not associated with increased risk of colorectal cancer even in the snoring group.
Lee (2017) [75]	Retrospective study plus case-control study. SDB was assessed by polysomnography. SDB was defined as an AHI ≥ 5.	163 consecutive patients who underwent overnight polysomnography and subsequent colonoscopy. For the case-control study, 195 age-, sex-, BMI-, and smoking-matched subjects without clinical symptoms of SDB who underwent colonoscopy were included.	-SDB was associated with an increased risk of having an advanced colorectal cancer for both, mild (AHI 5–14·9) and moderate-to-severe (AHI ≥ 15) SDB after adjusting for confounders (OR 14.09; 95% CI 1.55–127.83; and OR 14.12; 95% CI 1.52–131.25, respectively). -In the case-control study, patients with SDB had a three-fold greater risk of having an advanced colorectal tumor compared to the control group (OR 3.03; 95% CI 1.44–6.34).
**Prostate cancer**			
Chung (2016) [76]	Retrospective case-control study. Data were retrieved from a national health insurance database. SDB was diagnosed based on ICD-9 codes	1236 men with SDB and 4944 age-matched men without ICD-9 codes corresponding to SDB.	-The prevalence of prostate cancer was higher in the SDB than in the control group (0.97% vs. 0.40%, *p* = 0.013). -Men with SDB were more likely to have prostate cancer compared to the non-SDB men (adjusted OR 2.14, 95% CI 1.03-4.43).
**Kidney cancer**			
Vilaseca (2016) [77]	Retrospective study. Patients data, including Information on SDB, were retrieved from medical records and databases. SDB was based on self-report.	2579 patients who underwent radical or partial nephrectomy for clear cell renal cell carcinoma.	-The prevalence of self-reported SDB was 7%. - Self-reported SDB was associated with higher Fuhrman grade compared to those without SDB (adjusted OR 1.41, 95% CI 1.00–1.99). - Self-reported SDB was not associated with tumour size, metastasis-free survival or cancer survival.
**Pancreatic cancer**			
Dal Molin (2016) [78]	Retrospective study. Data were retrieved from medical records. SDB was diagnosed based on a positive polysomnography that was either directly available for review or reported in the medical records (the threshold to define SDB is not provided).	1031 patients who underwent surgical resection without neoadjuvant therapy for pancreatic ductal adenocarcinoma.	-Patients with SDB were significantly more likely to have lymph node-negative tumours than non-OSA cases (37.7% vs. 21.8%, *p* = 0.004). -SDB was an independent predictor of lymph node status (OR 0.51, 95%CI 0.27–0.96). -SDB, however, was not associated with greater cancer mortality (OR 0.89, 95% CI 0.65–1.24).
**Central nervous system cancers**			
Chen (2014) [79]	Retrospective case-control study. Data were retrieved from a National health institute database. SDB was diagnosed based on ICD-9 codes. 10-year follow-up period.	23,055 newly diagnosed cases of SDB and 69,165 age- and gender-matched individuals without ICD-9 codes corresponding to SDB.	-SDB was associated with higher incidence of primary central nervous system cancers compared to non-SDB individuals (adjusted HR 1.54, 95% CI 1.01–2.37). -SDB without surgical treatment was associated with higher incidence risk for primary CNS cancers than the comparison group (adjusted HR 1.83, 95% CI 1.23–2.74). -SDB patients who underwent surgical treatment for their sleep disorder had a similar risk of incident primary CNS cancers than the comparison group (adjusted HR 0.98, 95% CI 0.31–3.09).
Lymphoma (2020)[80]	Retrospective case-control study. Data were retrieved from a National health institute database. SDB was diagnosed based on ICD-10 codes. 4.8 ± 2.3 follow-up period.	198,574 newly diagnosed cases of SDB and 992,870 age- and gender-matched individuals without ICD-10 codes corresponding to SDB	-The incidence of non-Hodgkin lymphoma among patients with OSA was significantly higher than that among the controls (HR 1.40, 95%CI 1.16-1.69). - This incidence was higher in women than that in men (1.62 vs. 1.28), but difference by age were not observed.

**Table 4:** Proposed future studies.

Prospective longitudinal, multicentred studies focused on a single organ cancer type that incorporate polysomnographic assessments at the time of cancer diagnosis, track adherence to treatment of OSA, and include multi-omics and tumour heterogeneity characterization.
Longitudinal studies of large cohorts of patients diagnosed with OSA without cancer and matched controls and long-term surveillance for cancer.
Prospective cancer treatment outcomes of patients with and without OSA with site- and type-specific cancers.
Search for OSA-related specific cancer biomarkers of aggressiveness, treatment selection, and personalized outcomes.
In vitro studies of differential effects of variable sustained and intermittent hypoxia profiles on specific tumours and on a large repertoire of specific tumour variants that incorporate multicellular matrices and organoid-based cellular interactions.
Exploration of animal models of OSA and their effect on cancer immunosurveillance and formulation of novel preventive strategies.
Identification and development of novel cancer therapeutic targets related to intrinsically driven pathways related to sleep-disordered breathing.
